# 

**Published:** 1885-03

**Authors:** 


### MARCH, 1885.

### CONTENTS.

ORIGINAL PAPERS:— *Page.*

Discussion on Intestinal Obstruction at the Bristol Meeting of the Bath and Bristol Branch of the British Medical Association ... 1

CLINICAL RECORDS:—

Retention and Accumulation of Fæces. By J. E. SHAW, M.B. ... 43

Mangana Water. By E. LONG FOX, M.D. ... ... ... ... ... ... ... 47

Compound Comminuted Fracture of the Left Femur, with much Laceration of the Soft Parts and the Knee Joint freely opened. By CHARLES F. PICKERING, F.R.C.S. ... ... ... ... ... ... ... ... ... 48

Pendulous Fatty Tumour of Thigh. By SHIRLEY DEAKIN, F.R.C.S. Eng. ... ... ... ... ... ... ... ... ... ... ... ... ... ... ... ... 51

NOTES ON BOOKS ... ... ... ... ... ... ... ... ... ... ... ... ... ... 52

MEDICAL EXTRACTS ... ... ... ... ... ... ... ... ... ... ... ... ... ... 61

